# Network pharmacology-based investigation of potential targets of astragalus membranaceous-angelica sinensis compound acting on diabetic nephropathy

**DOI:** 10.1038/s41598-021-98925-6

**Published:** 2021-09-30

**Authors:** Youzi Dong, Quanlin Zhao, Yuguo Wang

**Affiliations:** 1 The First Clinical Medical College, Shandong University of Traditional Chinese Medicine, Jinan, Shandong China; 2 Division of Comprehensive Internal Medicine, Shandong University of Traditional Chinese Medicine Affiliated Hospital, Jinan, Shandong China; 3 Department of Traditional Chinese Medicine, Sixth Medical Center of Chinese PLA General Hospital, Beijing, China

**Keywords:** Diabetes complications, Data mining

## Abstract

To explore the mechanism of the Astragalus membranaceous (AM)-Angelica sinensis (AS) compound in the treatment of diabetic nephropathy (DN) we used network pharmacology and molecular docking. Screen the components and targets of the AM-AS compound in the TCMSP and the BATMAN-TCM, and establish a component-target interaction network by Cytoscape 3.7.2. After searching relevant targets of DN in related databases, the common targets of the AM-AS compound and DN were obtained by comparison. Gene ontology (GO) analysis and Kyoto Encyclopedia of Gene and Genome (KEGG) pathway enrichment analysis were performed through David database. Molecular docking was performed by PyMoL2.3.0 and AutoDock Vina software. After screening, 142 main targets of the AM-AS compound in the treatment of DN have been identified. Target network was established and the topology of PPI network was analyzed. KEGG pathway enrichment analysis shows that these targets are related to apoptosis, oxidative stress, inflammation, insulin resistance, etc. Molecular docking shows that the target proteins have good combinations with the main active components of the AM-AS compound. AM-AS compound may treat DN by acting on VEGFA, TP53, IL-6, TNF, MARK1, etc., and regulate apoptosis, oxidative stress, inflammation, glucose, and lipid metabolism processes. The in vivo study results suggest that AM-AS compound can significantly reduce the FBG level of diabetic rats, increase the level of INS, improve renal functions, reduce urinary proteins, inhibit glycogen deposition, granulocyte infiltration and collagen fiber proliferation in renal tissue, and restrain the progress of DN. In vivo study combined with network pharmacology and molecular docking methods provides new ideas for the pathogenesis and treatments of DN.

## Introduction

Recent epidemiological studies show that diabetes currently affects more than 440 million people worldwide. As of 2013, about 11 percent of China's population had diabetes, and a large portion remained undiagnosed^1^. About 24 million people in the United States have diabetes, with type 2 diabetes(T2DM) accounting for the vast majority^2^. T2DM is a chronic multi-system disease. Long-term persistent hyperglycemia affects the microvascular system, causing diabetic nephropathy(DN), retinopathy, neuropathy, and other complications^3^. In a recent population-based study, the prevalence of DN was reported to be 30.9% in patients with diabetes, in which substandard blood glucose was an important determinant^4^. DN is one of the major causes of end-stage renal disease (ESRD), damage caused by diabetes can affect almost all structures of the kidney, and progress much faster than non-DN patients, and the long-term prognosis is bad. Clinical treatment focuses on controlling blood sugar and blood pressure, improving renal function, and reducing urine protein^5^. Traditional Chinese medicine(TCM) has good effects in improving diabetic symptoms, preventing and treating DN and other complications, and improving patients' quality of life. Our previous studies have found that the medicine for invigorating qi and promoting blood circulation can lower blood sugar and delay the progress of apoptosis caused by high glucose^6,7^.

Network pharmacology is a new discipline based on the theory of systems biology, which analyzes the network of biological systems and selects specific signal nodes to design multi-target drug molecules^8^. The predictability is a characteristic of network pharmacology, which emphasizes the multi-channel regulation of signaling pathways, improves the therapeutic effect of drugs, reduces the toxic and side effects, and thus improves the success rate of clinical trials of new drugs and saves the cost of drug research and development^9^. Molecular docking is a method of drug design based on the characteristics of the receptor and the interaction between the receptor and the drug molecule, a theoretical simulation method for predicting binding patterns and affinity of molecular interactions. In recent years, molecular docking has become an important technology in the field of computer-aided drug research^10^. In this study, we use network pharmacology methods combined with molecular docking to establish the AM and AS compound-DN-targets interaction network to provide a basis for further exploration of pathogen and the interpretation of the mechanism of the AM-AS compound in treating DN.

## Methods

### Data preparation

The Traditional Chinese Medicine Systems Pharmacology Database and Analysis Platform (TCMSP^11^, http://tcmspw.com/tcmsp.php) is a unique Chinese herbal medicine system pharmacology platform, which highlights the role of system pharmacology in TCM. We used this database to obtain the relationship between the AM-AS compound, targets, and DN. We retrieved all the components of the AM-AS compound from the TCMSP, and the components’ oral bioavailability (OB) ≥ 30% and drug-likeness (DL) ≥ 0.18 is used as the screening condition^11^. Enter the target name in the UniProt^12^ database (https://www.uniprot.org/). Standardize the genes and record. A Bioinformatics Analysis Tool for Molecular mechANism of Traditional Chinese Medicine (BATMAN-TCM^13^, http://bionet.ncpsb.org/batman-tcm/) is the first online bioinformatics analysis tool specially designed for the research of the molecular mechanism of TCM. BATMAN-TCM will first predict potential targets for each component, and then perform functional analyses on the above targets, AM and AS compound-targets-DN association network will be shown. These functions contribute to the understanding of the “multi-component, multi-target and multi-pathway” combinational therapeutic mechanism of the AM-AS compound and provide clues for the following experimental validation. We searched AM and AS in BATMAN-TCM. The search conditions were Score cutoff ≥ 20, P-value cutoff < 0.05^14^, and the components comply with OB ≥ 30% and DL ≥ 0.18. Standardize the genes and import all the above results into EXCEL. Merge predicted targets of the two databases, establish the AM and AS compound-component-targets database, and construct the network diagram by Cytoscape 3.7.2 software.

Search disease targets in the Comparative Toxicogenomics Database (CTD, http://ctdbase.org/), the Online Mendelian Inheritance in Man database (OMIM, https://www.omim.org/), and the Human Gene Database (GeneCards, https://www.genecards.org/) with the keyword of "diabetic nephropathy".Take the intersection with the AM-AS compound targets.

### Network construction

We collected these targets and got the AM and AS compound-targets-DN interaction network. The core targets were imported into STRING^15^ (https://string-db.org/). Predicted protein–protein interactions (PPI) were got with the condition of confidence score > 0.9. The above results were imported into Cytoscape 3.7.2 software for visualization, and the MCODE plug-in was used to screen important PPI network modules. The GO function (cell function, molecular function, and biological function) analysis and KEGG pathway enrichment of co-action targets were conducted by the BINGO plug-in and the David database(https://david.ncifcrf.gov/). P < 0.05 indicates significant differences^16^.

### Molecular docking

The top 5 target proteins in the PPI network were selected for molecular docking. We download the PDB format file of the 3D structure of the targets in the RCSB PDB (https://www.rcsb.org/) database and download the mol2 format file of the 3D structure of the core active component from the TCMSP database. DN clinical recommended drugs (Irbesartan, Canagliflozin, benazepril, captopril) were searched in DRUNGBANK^17^ (https://go.drugbank.com/). The SDF format files of the 2D structure of the drugs were downloaded from the DRUNGBANK database and were converted to the mol2 format file by Open Babel 3.1.1. Molecular docking with the main active components of the AM-AS compound recommended drugs, and core target proteins were performed by PyMoL 2.3.0 and AutoDock Vina^18^. The binding activity is evaluated by the lowest binding efficiency.

### In vivo study

#### Materials and equipment

Streptozotocin (STZ) was purchased from Sigma company, the SGLT-2 inhibitor(SGLT-2i) canagliflozin (Invokana, 100 mg/tablet × 10 tablets, H20170375) was obtained from Janssen-Cilag S.p. A.. AM and AS granules were purchased from the Affiliated Hospital of the Shandong University of Traditional Chinese Medicine (specifications: 10 g/package). Rat insulin (INS) ELISA kit, rat β2-microglobulin (β2-MG) ELISA kit, and rat microalbuminuria (MAU) ELISA kit were purchased from Wuhan Huamei Biological Engineering Co., Ltd. Rat blood urea nitrogen (BUN) mensuration reagent kit, serum creatinine (Scr) mensuration reagent kit and glycosylated serum proteins (GSP) mensuration reagent kit were purchased from Changchun Huili biotech Co., Ltd. Ultrasonic cell crusher (JY92-IIn, Ningbo Xinzhi Biotechnology Co., Ltd.), automatic biochemical analyzer (Chemray 240, Rayto Co., Ltd.), enzyme label detector (Epoch, BioTeK Company) and microscopes (NIKON Eclipse ci, MADE IN JAPAN) were provided by the laboratory of Affiliated Hospital of Shandong University of Traditional Chinese Medicine.

#### Animals

20 SPF male Sprague–Dawley (SD) rats were provided by Jinan Pengyue Experimental Animal Breeding Co., Ltd. The whole study followed the requirements of relevant guidelines^19,20^ and was approved by the requirements of the Experimental Animal Ethics Committee of the Affiliated Hospital of Shandong University of Traditional Chinese Medicine(AWE-2019-007). The rats were divided randomly into 4 groups, 5 in each group, including blank control (BC) group, negative control (NC) group, astragalus membranaceous-angelica sinensis compound (AM-AS) group, and canagliflozin (SGLT-2i) group. The latter two are positive controls(PC). The rats of the BC group were fed with a normal diet, and the rests were fed with high-fat and high-sugar diets for 6 weeks. After fasting for 12 h, STZ (30 mg kg^−1^) was injected intraperitoneally. Rats with 3 consecutive times fasting glucose levels ≥ 16.7 mmol L^−1^ 72 h after the injection were considered diabetic and were included in the study^21,22^.

#### Experimental design

The rats of each group were given intragastric administration on the second day after the successful establishment of the model. The AM-AS group was given 1 g mL^−1^ AM-AS compound solution (5.40 g kg^−1^ day) by stomach irrigation. The SGLT-2i group was given a canagliflozin solution, and the dose was converted according to the relevant guidelines^23,24^. During the medication period, the BC group was given a normal diet, and the rests were given high-fat and high-sugar diets. The general conditions of rats were observed daily, and the bodyweight (WB) and FBG were measured weekly. After 12 weeks of intragastric administration, the rats were killed and the renal tissues were separated quickly. Paraffin sections were made and were treated with HE staining and Masson staining. The pathological and ultrastructural morphological observations were carried out, and the degree of renal tissue damage, fibrous connective tissue hyperplasia, and inflammatory cell infiltration were compared. One-way ANOVA was used to analyze the data by IBM SPSS 19.0. *P* < 0.05 was considered statistically significant.

## Results

### Data preparation

**Figure 1:**
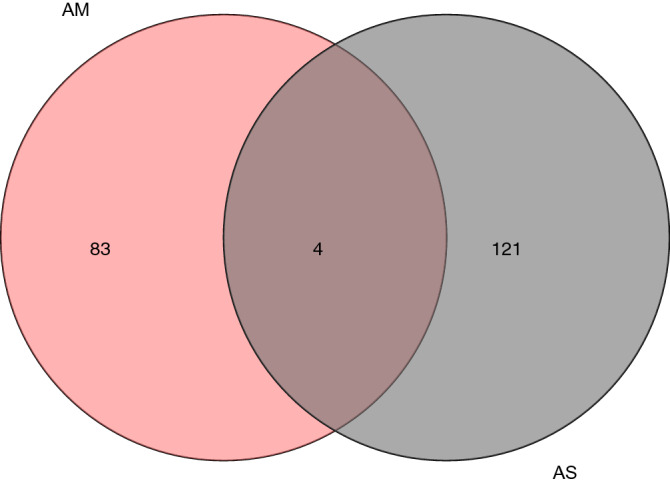
Venn of compounds' screening. After preliminary screening, there are 87 components of AM, 125 components of AS, and 4 components shared by both AM and AS.

**Figure 2:**
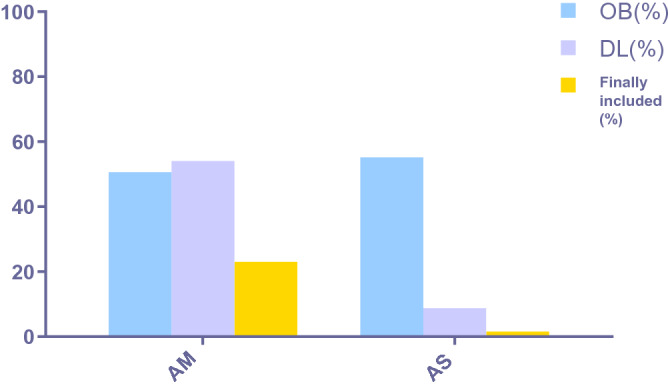
Proportion of active components included. Among the components of AM, 50.57% meet OB ≥ 30%, 54.02% meet DL ≥ 0.18, and the final inclusion ratio is 22.99%. Among the components of AS, 22.99% meet OB ≥ 30%, 8.80% meet DL ≥ 0.18, and the final inclusion ratio is 1.60%.

**Figure 3:**
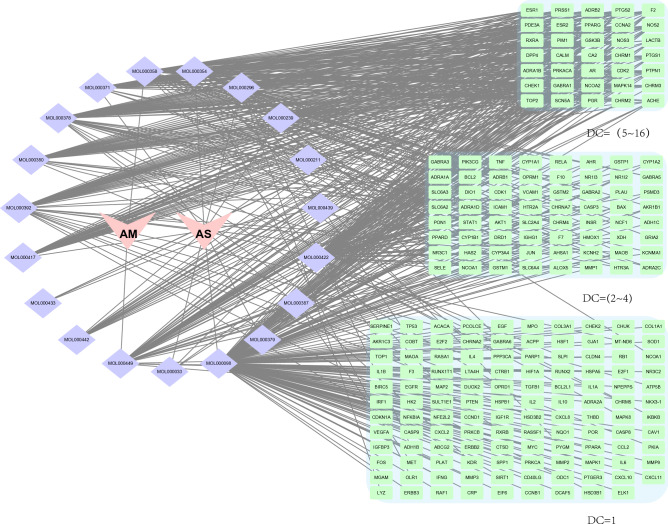
Interaction Nnetwork of AM&AS compound-components-targets. Red inverted triangles represent AM and AS, purple diamonds represent active components, and green rectangles represent target genes, which are grouped by degree centrality (DC) values.

After combining the results we searched in TCMSP and BATMAN-TCM, 208 components of the AM-AS compound were obtained (Fig. 1). These components were screened with the condition of OB ≥ 30% and DL ≥ 0.18 and the percentage of active components finally included were calculated (Fig. 2). After further screening, 22 components were obtained (Table 1), including 2 components of AS and 20 components of AM. There are 634 core prediction targets, including 93 of AS and 541 of AM, and 58 common targets(Fig. 3). 1993 disease targets were searched in TTD, DrugBank, DisGeNET, CTD, OMIM, and GeneCards databases, which related closely with DN.

**Table 1:** Components of AM-AS compound (top 10 of OB value).

Mol_ID	Component name	OB (%)	DL
MOL000398	Isoflavanone	109.99	0.3
MOL000378	7-O-Methylisomucronulatol	74.69	0.3
MOL000392	Formononetin	69.67	0.21
MOL000433	Fa	68.96	0.71
MOL000438	(3R)-3-(2-Hydroxy-3,4-dimethoxyphenyl)chroman-7-ol	67.67	0.26
MOL000380	(6aR,11aR)-9,10-Dimethoxy-6a,11a-dihydro-6H-benzofurano[3,2-cchromen-3-ol	64.26	0.42
MOL000211	Mairin	55.38	0.78
MOL000371	3,9-di-O-Methylnissolin	53.74	0.48
MOL000239	Jaranol	50.83	0.29
MOL000354	Isorhamnetin	49.6	0.31

**Figure 4:**
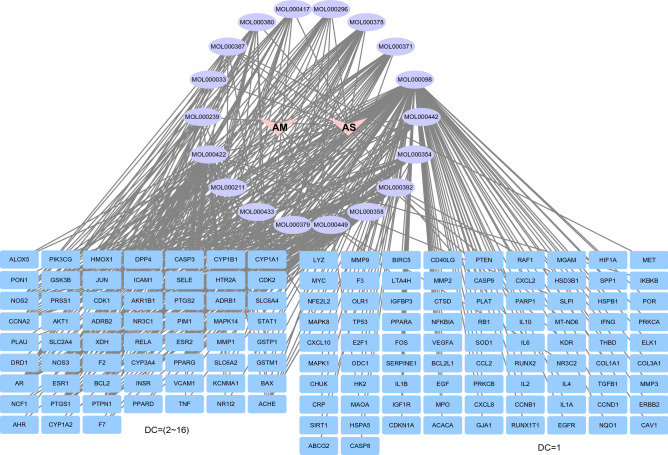
Interaction network of AM&AS compound- DN-targets. The red triangles represent AM and AS, the purple circles represent the active components, and the blue rectangles represent the target genes of the AM-AS compound acting on DN, which are divided by degree centrality (DC) values. The number of edges connected to the node in the network represents the degree of freedom.

142 common potential targets of AM and AS compound-DN were obtained by further comparing and screening, which correspond to 18 active components (Fig. 4). There are 14 components connect with more than 10 targets, including MOL000098 quercetin (122), MOL000422 kaempferol (51), MOL000354 isorhamnetin (27), MOL000358 β-sitosterol (27), MOL000449 bean sterols (23), MOL000371 3,9-dioxymethyl nisolin (21), etc. These components may play an important role in the pathogenesis and treatment of DN. There are 18 components’ target number ≥ 3, indicating the complexity of the mechanism in treating DN with the AM-AS compound.

### Network construction

**Figure 5:**
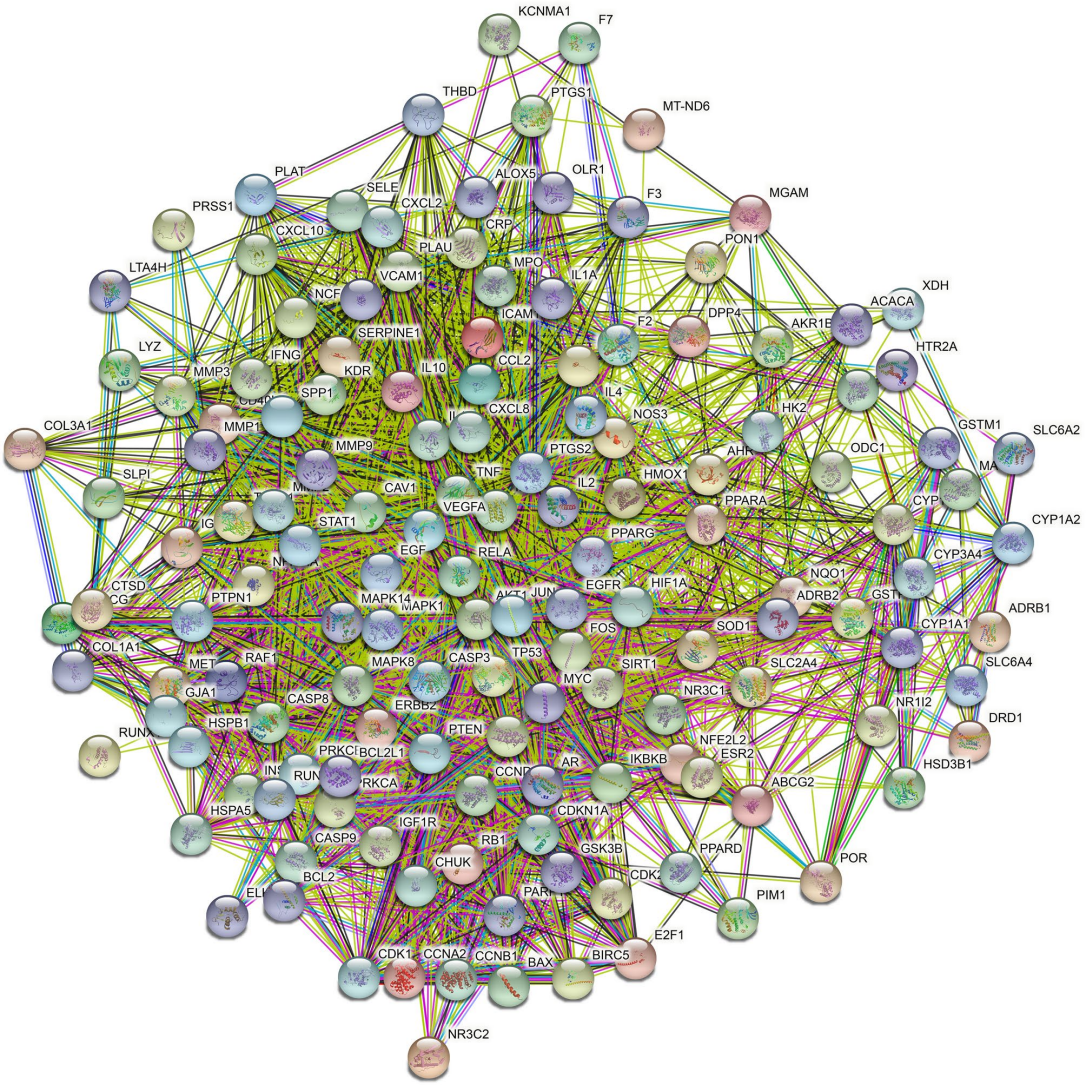
PPI network. Network nodes represent proteins: colored nodes represent query proteins and the first shell of interactors; white nodes represent the second shell of interactors; empty nodes represent proteins of unknown 3D structure; filled nodes represent some 3D structure that is known or predicted. Edges represent protein–protein associations: the light blue edges represent from curated databases; the fuchsia edges represent experimentally determined; the green edges represent gene neighborhood; the red edges represent gene fusions; the dark blue edges represent gene co-occurrence; the light green edges represent text mining; the black edges represent co-expression; the light purple edges represent protein homology. The thickness of the line in the figure represents the strength of the force.

**Figure 6:**
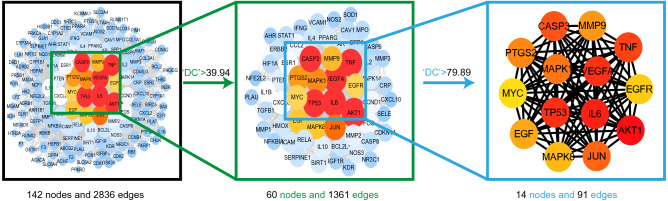
Core targets topology. The figure shows the topology of the PPI network. The color of the targets changes from blue to red according to the DC value, and red represents the core targets. The black box shows all the targets, the green box shows the targets whose DC value is greater than 1 times the median, and the blue box shows the targets whose DC value is greater than 2 times the median, which are the core targets of this study.

**Figure 7:**
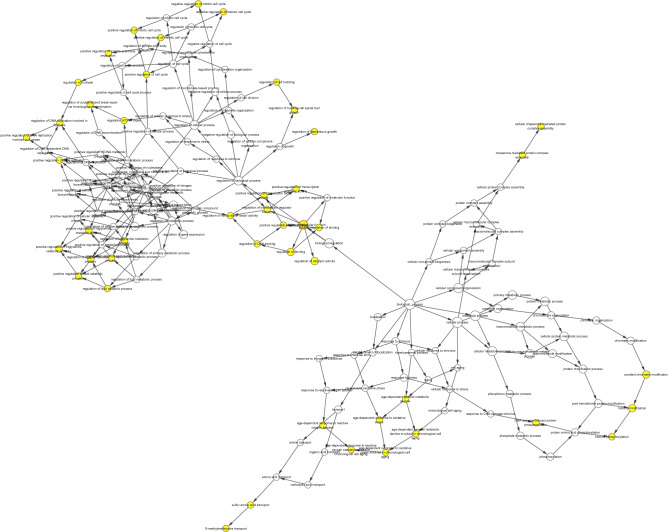
Functional enrichment analysis.

142 common targets were imported into the STRING database, as shown in Fig. 5. The PPI network of the AM-AS compound against DN was gathered by Cytoscape 3.7.2. We performed cluster analysis of the PPI network, extracted the targets with degree Centrality (DC) values greater than 1 times the median or 2 times the median, and screened out the top 14 targets in the number of nodes by CytoHubba plug-in (Fig. 6). GO analysis results show that 502 biological processes (BP), 45 cell components (CC), 96 molecular functions (MF) were obtained in the David database. Sorted by the degree of significance, the BP of AM and AS compound-DN-targets is significantly enriched in drug response, negative regulation of apoptosis process, positive regulation of RNA polymerase II promoter transcription, deficiency Oxygen response, positive regulation of gene expression, inflammatory response, aging, etc. MF is mainly enriched in enzyme binding, protein binding, transcription factor binding, protein isomerization activity, RNA polymerase II transcription factor activity, steroid hormone receptor, protein kinase binding, cytokine activity, etc. CC is mainly enriched in the extracellular space, extracellular zone, cytoplasm, mitochondria, fossa, nucleoplasm, extracellular matrix, plasma membrane, etc. (Table 2). Further analysis by the BINGO plug-in is shown in Fig. 7.

**Table 2:** GO analysis of core targets (top 10).

Category	Gene function	Count	P-value
BP	Response to drug	30	1.05 × 10^–22^
BP	Negative regulation of apoptotic process	34	4.97 × 10^–22^
BP	Positive regulation of transcription from RNA polymerase II promoter	42	1.68 × 10^–18^
BP	Positive regulation of transcription, DNA-templated	32	2.31 × 10^–18^
BP	Response to hypoxia	20	2.55 × 10^–16^
BP	Positive regulation of gene expression	23	3.38 × 10^–16^
BP	Cellular response to hypoxia	16	2.27 × 10^–15^
BP	Inflammatory response	25	8.71 × 10^–15^
BP	Response to estradiol	15	2.50 × 10^–14^
BP	Aging	18	3.54 × 10^–14^
MF	Enzyme binding	32	5.35 × 10^–24^
MF	Protein binding	120	1.28 × 10^–16^
MF	Identical protein binding	32	7.18 × 10^–14^
MF	Transcription factor binding	21	2.29 × 10^–13^
MF	Protein heterodimerization activity	20	1.01 × 10^–8^
MF	RNA polymerase II transcription factor activity, ligand-activated sequence-specific DNA binding	8	1.58 × 10^–8^
MF	Steroid hormone receptor activity	9	1.86 × 10^–8^
MF	Protein homodimerization activity	24	3.29 × 10^–8^
MF	Protein kinase binding	17	9.26 × 10^–8^
MF	Cytokine activity	12	2.45 × 10^–7^
CC	Extracellular space	48	1.42 × 10^–19^
CC	Membrane raft	16	5.96 × 10^–11^
CC	Extracellular region	39	2.06 × 10^–10^
CC	Cytosol	58	3.83 × 10^–10^
CC	Mitochondrion	32	1.96 × 10^–8^
CC	Caveola	9	3.46 × 10^–8^
CC	Nucleoplasm	45	1.13 × 10^–6^
CC	Extracellular matrix	13	3.09 × 10^–6^
CC	Plasma membrane	56	6.49 × 10^–6^
CC	Nucleus	67	9.72 × 10^–6^

**Figure 8:**
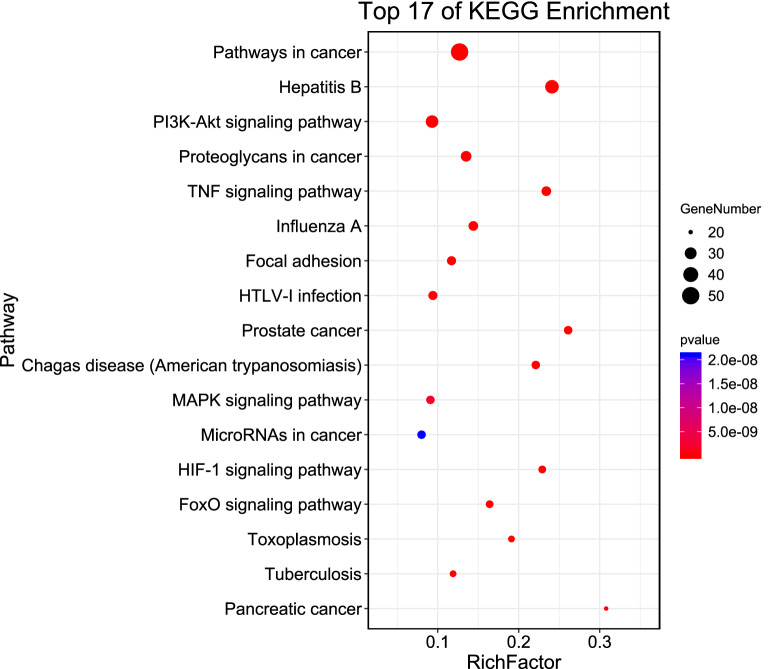
Pathways of AM-AS compound against DN (count > 20).

120 KEGG enrichment pathways related to DN were obtained with the condition of P < 0.05. Sorted by the target number of the pathway, we select pathways with the number of genes > 20 (Fig. 8). Cancer pathway, PI3K-Akt signaling pathway, proteoglycan in cancer, hypoxia-inducible factor-1(HIF-1) signaling pathway, and tumor necrosis factor signaling pathway are included. There are 43 disease-related pathways, including type 1 diabetes(T1DM), T2DM, pancreatic cancer, rheumatoid arthritis, hepatitis B, hepatitis C, non-alcoholic fatty liver, cancer pathway, tuberculosis, etc. Inflammation-related pathways include TNF signaling pathway, nuclear transcription factor-κB (NF-κB) signaling pathway, etc. In addition, there are the HIF-1 signaling pathway, insulin resistance pathway, cyclic adenosine monophosphate (cAMP) signaling pathway, etc. AM-AS compound may act on these pathways against DN.

### Molecular docking

**Figure 9:**
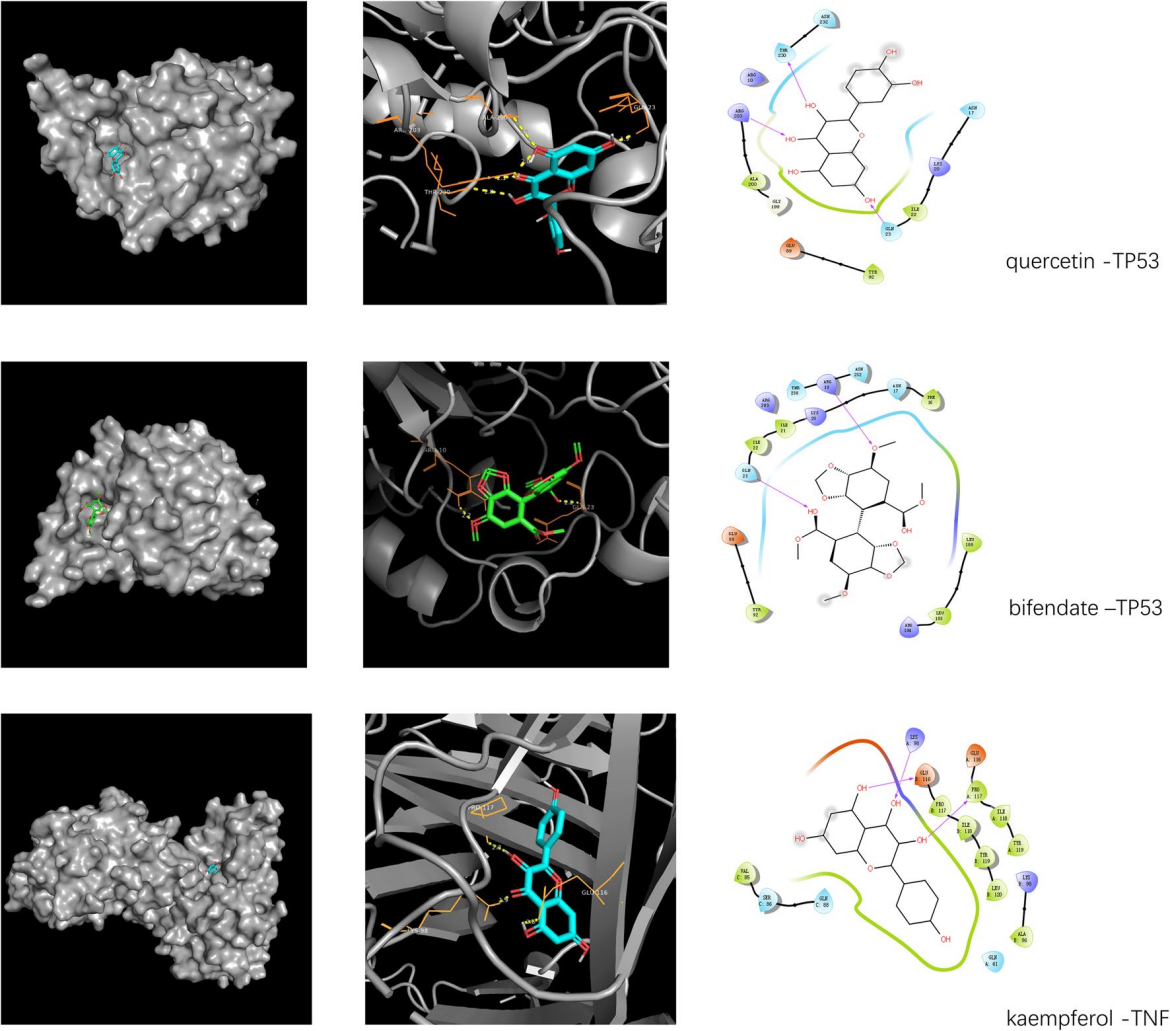
Molecular docking patterns. The yellow lines represent the hydrogen bond interaction force, which is the main force promoting molecule binding with the active site.

Among the 18 core active components, we select the top 5 target proteins (IL-6, TP53, VEGFA, TNF, MARK1) in the PPI network whose degree > 10 in the "AM and AS compound- DN-targets" interaction network. The core active components (quercetin, kaempferol, isorhamnetin, etc.) were molecularly docked by AutoDock Vina, as shown in Table 3. The absolute values of the docking score indicate the affinity of the components with the targets and the stability of the conformation. The absolute value greater than 4.25 indicates a certain binding activity, greater than 5.0 indicates a good binding activity, and greater than 7.0 indicates a strong binding activity^25^. The molecular docking results showed that the binding ability of quercetin, kaempferol, and isorhamnetin to TP53 was stronger, and the binding energy of each active component was stronger than that of captopril. The binding energy of β-sitosterol and stigmasterol to IL-6 is higher than that of benazepril, captopril, and irbesartan. The binding ability of each active component with VEGFA is poor but stronger than captopril. Quercetin, formononetin, mullein, kaempferol, and TNF have stronger affinity than canagliflozin, benazepril, and captopril. Comprehensive analysis shows that the docking scores of quercetin with TP53 and stigmasterol with IL-6 have the largest absolute value among the five targets. The targets TP53, IL-6, VEGFA, TNF, and MARK1 have the strongest affinity with quercetin, stigmasterol, and kaempferol. The specific binding patterns of the target proteins and components were processed and optimized by PyMoL2.3.0 (Fig. 9).

**Table 3:**
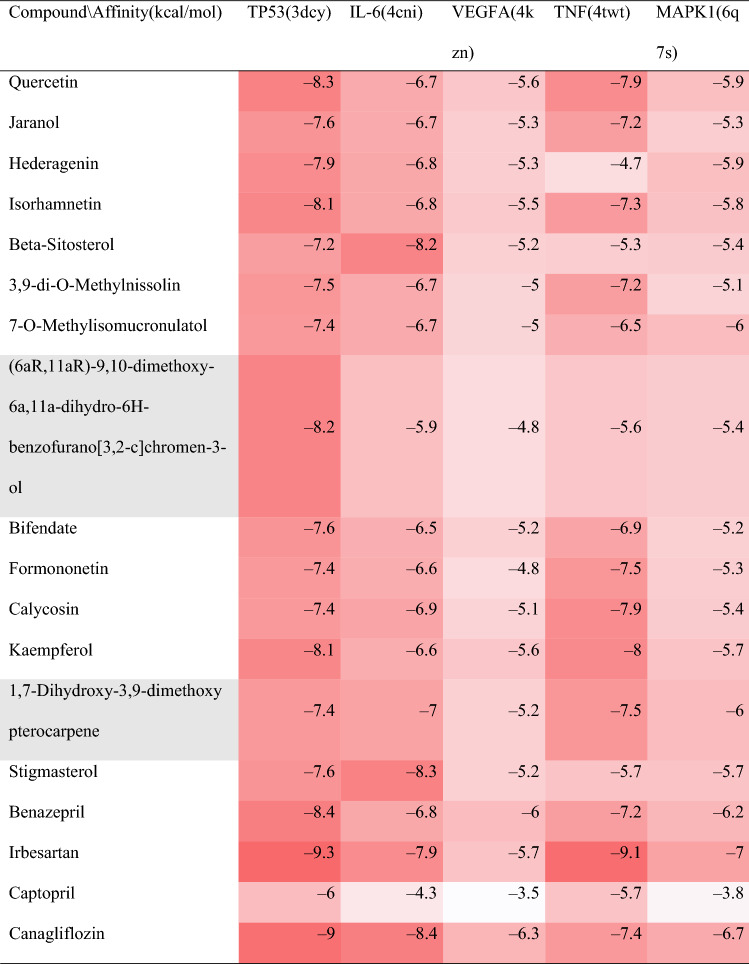
Docking results of core target proteins and core active components.

### In vivo study

#### General conditions

The levels of FBG and WB relief of rats in the NC group were higher than those in the BC group (*P* < 0.01). Compared with the NC group, the general conditions of the positive control groups (AM-AS group & SGLT-2i group) were significantly improved (*P* < 0.01). The comparison between the two groups showed that the effect of reducing FBG and interfering with WB in the SGLT-2i group was more significant than that in the AM-AS group (*P* < 0.05), as shown in Table 4.

**Table 4:** General conditions of rats (*X* ± *S*).

Group	*n*	FBG (mmol L^−1^)	WB (mg)
BC	5	4.08 ± 0.35	463.00 ± 16.06
NC	5	28.46 ± 1.41^△△^^,^*	361.40 ± 14.74^△△^^,^*
AM-AS	5	19.32 ± 1.26^△△^^,^^##^^,^*	396.20 ± 18.86^△△^^,^^##^^,^*
SGLT-2i	5	17.08 ± 0.84^△△^^,^^##^	417.40 ± 11.28^△△^^,^^##^

^△△^P < 0.01 vs. BC, ^##^P < 0.01 vs. NC, *P < 0.05 vs. SGLT-2i.

#### Medical laboratory examination

NC group and BC group have apparent disparities in GSP, BUN, Scr, INS (Corrected OD value), β2-MG, and MAU (*P* < 0.01). Compared with the NC group, the AM-AS group has huge ameliorations in BUN, INS, β2-MG, and MAU (*P* < 0.01), and has many improvements in GSP and Scr (*P* < 0.05). All of the above was more significantly different in the SGLT-2i group than in the AM-AS group (*P* < 0.01), especially in reducing INS, β 2-MG, and MAU. Both positive-control groups have great effects on regulating BUN, GSP, and Scr, as shown in Tables 5 and 6.

**Table 5:** Medical laboratory examination of serum (*X* ± *S*).

Group	*n*	GSP (mmol∙L^-1)^	BUN (mg∙dl^-1^)	Scr (umol∙L^-1^)	INS (nIU∙mL^-1^)
BC	5	1.60 ± 0.18	15.15 ± 1.97	15.75 ± 3.32	0.57 ± 0.06
NC	5	2.77 ± 0.35^△△^^,^*	31.50 ± 3.53^△△^^,^*	32.27 ± 4.16^△△^^,^*	0.21 ± 0.10^△△^^,^*
AM-AS	5	2.27 ± 0.21^△△^^,^^#^	22.43 ± 2.65^△△^^,^^##^	20.80 ± 3.17^△,##^	0.34 ± 0.03^△△^^,^^##^^,^*
SGLT-2i	5	1.96 ± 0.32^##^	19.43 ± 2.27^△,##^	18.21 ± 2.38^##^	0.45 ± 0.08^△,##^

^△△^P < 0.01 vs. BC, ^△^P < 0.05 vs. BC, ^##^P < 0.01 vs. NG, ^#^P < 0.05 vs. NC, *P < 0.05 vs. SGLT-2i.

**Table 6:** Medical laboratory examination of urine(*X* ± *S*).

Group	*n*	β2-MG(µg∙ml^-^1)	MAU(µg∙ml^-1^)
BC	5	0.55 ± 0.05	0.34 ± 0.06
NC	5	1.05 ± 0.07^△△^^,^*	0.88 ± 0.09^△△^^,^*
AM-AS	5	0.81 ± 0.03^△△^^,^^##^^,^*	0.66 ± 0.08^△△^^,^^##^^,^*
SGLT-2i	5	0.68 ± 0.08^△△^^,^^##^	0.45 ± 0.04^##^

^△△^P < 0.01 vs. BC, ^##^P < 0.01 vs. NC, *P < 0.05 vs. SGLT-2i.

#### Pathological observation

**Figure 10:**
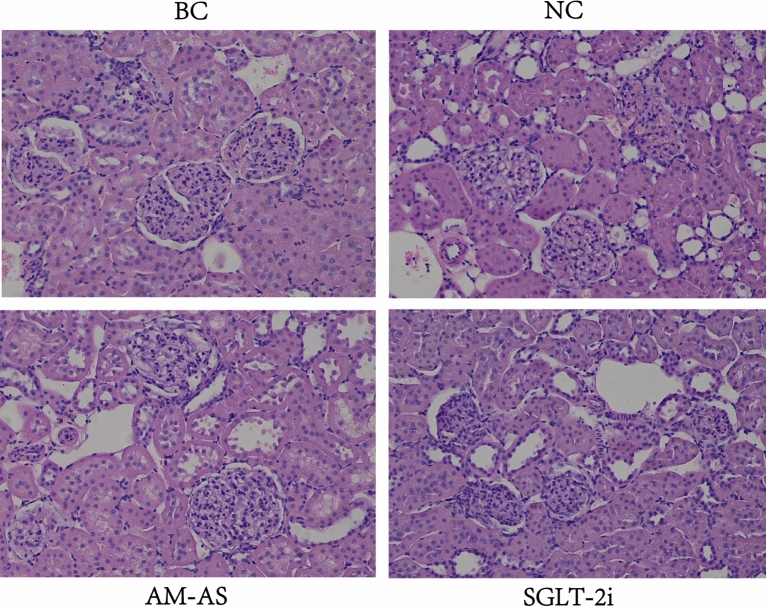
Renal tissue cells of rats (HE × 200).

Through HE staining, the structure of glomeruli and tubules of rats in the BC group was distinct, the arrangements of renal tubules were compact, the glomerular capillaries were demarcated, and a small amount of granulocyte infiltration could be observed visibly. In the NC group, it can be seen that a small number of renal tubules dilated and renal tubular epithelial cells narrowed and vacuolated. The nucleus of renal tubular epithelial cells was hyperchromatic, suspected to be glycogen deposition. The glomerular capillaries were distinct in the visual field, granulocytes were infiltrated, and some glomerular capillaries were congested. In the AM-AS group, the renal tubules dilated, the epithelial cells of renal tubules narrowed, and the structures of glomerular capillaries did not change significantly. In the SGLT-2i group, the renal tubules were tightly arranged, a few renal tubules were dilated, the renal tubular epithelial cells were flat, and the glomerular capillary structure had no obvious changes. The above two groups were improved after treatments, suggesting that AM-AS compound can inhibit the vacuolation of renal tubular epithelial cells, reduce glycogen deposition and granulocyte infiltration, and delay the progression of DN (Fig. 10).

**Figure 11:**
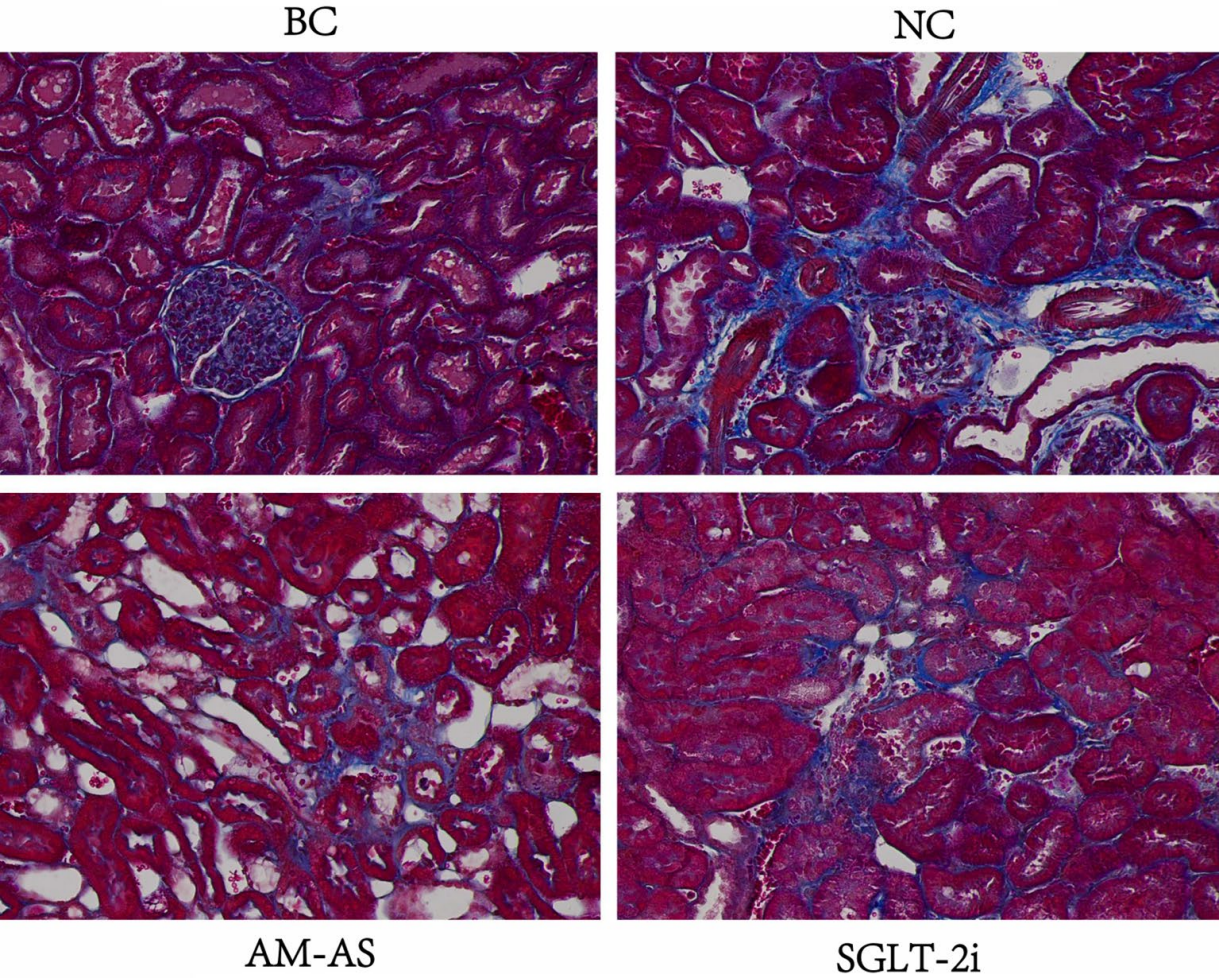
Renal tissue cells of rats (Masson × 200).

Masson staining showed that there were no obvious proliferations of blue-stained collagen fibers in the kidney tissue of rats in the BC group. In the NC group, there were more blue-stained collagen fibers around blood vessels and local renal tubulointerstitium. The increase of blue-stained collagen fibers could be seen in the local renal tubulointerstitium of both the AM-AS group and SGLT-2i group, suggesting that the AM-AS compound can inhibit the production of collagen fibers and slow down the process of renal fibrosis of DN(Fig. 11).

## Discussion

According to the clinical manifestations, diabetes mellitus (DM) is the same as “Xiaoke” in TCM, and the earliest records of etiology and pathogenesis can be traced back to "*Neijing*". As "*Lingshu*" records: “People who are weak in the five viscera are prone to ‘Xiaoke’.” It has been realized that insufficiency of the innate endowment is an important internal factor causing diabetes. "*Suwen*" records: “Patients who often eat luscious and fatty foods may produce ‘internal heat’. And sweetness can result in abdominal bloating, which affects the abnormal spleen and stomach mobilization. Then damp-heat overflows and diabetes occurs.” Irregular diet, gastrointestinal heat buildup, and consumption of yin are considered to be the main pathogenesis. “Xiaoke” is divided into multiple syndrome types according to different disease locations, pathogenesis, and symptoms. Among them, the syndrome of insufficient kidney yin corresponds to DN, which known as "Xiaxiao"."*Sheng Ji Zong Lu*" records: "The cause of this disease is the deficiency of essence and blood, which results in insufficient of kidney yin. Patients are often thirsty for drinking water, which is transported down to the bladder, resulting in a lot of urine. Other symptoms include calf muscle wasting and sore joints. Therefore, it was also named 'Xiaoshen'". The TCM pathogenesis of DN is based on deficiency in origin(Spleen and Kidney) and excess in superficiality(blood stasis, water dampness, turbidity). Early lesions are qi and yin injuries, liver and kidney deficiency, blood stasis blocking. Then yin deficiency causes yang deficiency, spleen, and kidney deficiency, resulting in water retention and overflowing to the skin. The deficiency of qi and yang can make blood flow unfavorable and aggravate stasis. In the end-stage, the water stays and gathers due to the decline of the kidney yang. Critical symptoms occur as a result of damp toxins, which attack the stomach and heart. Therefore, spleen deficiency is the key pathogen of DN; lack of spirit qi is the root pathogen of DN, and blood stasis is involved in the whole course of DN.

The principle of TCM treating DN is to " strengthening the body resistance to eliminate pathogenic factors" and "balance the yin and yang". In clinical treatment, the urgency of the syndromes and symptoms must be distinguished. Treat symptomatically in acute cases, and remove the primary disease in chronic cases. “*Jin Gui Yao Lue*” records effective prescriptions such as “White Tiger Plus Ginseng” decoction and “Shenqi” pills, which have been highly praised by clinical physicians so far. "*Tai Ping Sheng Hui Fang*" contains dry Rehmannia prescription and “Shen Li Yuan” prescription. "*Sheng Ji Zong Lu*" proposed “Liu Wei Di Huang” pills, “Zi Cui” decoction, “Zuo Gui” decoction, and many other famous prescriptions. Zhang Xichun, a famous doctor in the Qing dynasty, reused AM and Chinese yam in the "Yuye” decoction, which meant to invigorate the qi and invigorate the spleen to cure diabetes.

A large number of studies have concluded that the blood system of DM patients is usually hypercoagulable. Long-term hypercoagulability is an important cause of impaired renal function, and DM patients have higher incidences of thrombosis^26^. When proteinuria occurs in DN, the activity of clotting factors in plasma is higher^27^. Therefore, we believe that DN occurs because of the long course of diabetes, leading to the deficiency of the spleen and kidneys, and the inability to transport water and dampness, which causes the overflow. Patients often have "blood stasis" symptoms, such as hyperlipidemia, hyperviscosity, microcirculation disorders, etc. Therefore, the treatment should be based on strengthening the spleen and kidney, replenishing spirit, and promoting blood circulation, and removing blood stasis. Treating both symptoms and primary disease can often receive a satisfactory curative effect. AM-AS compound is one of the representative Chinese herbal medicine pairs, which has a large number of theoretical foundations and wide clinical applications. Because it can not only form a party on its own but also can be used as a basic drug pairing prescription, it has been valued by the doctors of the past generations. The prescription principle of the AM-AS compound is the distribution theory of qi and blood, which includes nourishing qi and blood, replenishing qi with invigorating blood, and replenishing qi with promoting blood circulation. "Qi is the commander of blood, and blood is the mother of qi", which means that sufficient spleen qi can transport and transform the essence into the lungs, and sufficient lung qi can combine with heart qi to generate blood, which can spread throughout the body. That combination also implies the mutual use of yin and yang. Qi belongs to yang and blood belongs to yin. The mutual nourishment of yin and yang facilitates the vitality of qi and metaplasia of blood. AM-AS compound is one of the common clinical prescriptions. Therefore, we studied the AM-AS compound by network pharmacology method and performed preliminary verification by molecular docking.

Through the analysis of the AM and AS compound-DN-targets network, we found that the main active components of the AM-AS compound treating DN included quercitrin, kaemferol, isorhamnetin, βsitosterol, stigmasterol, etc. By topological analysis of the PPI network, we found that the core targets were TNF, IL-6, MAPK1, MAPK8, AKT1, JUN, etc. Quercetin mainly affects IL-6, VEGFA, and TP53. Kaempferol mainly affects TNF. GO analysis showed that the BP of AM-AS compound against DN mainly concentrated in the direction of apoptosis, positive regulation of RNA polymerase II promoter transcription, hypoxia response, inflammatory response, etc. The main pathways obtained by KEGG analysis include PI3K-Akt signaling pathway, TNF signaling pathway, NF-κB signaling pathway, MAPK signaling pathway, HIF-1 signaling pathway, FOXO signaling pathway, etc. 31 pathways contain IL-6, such as HIF-1 signaling pathway and insulin resistance pathway, etc. 104 target genes connect closely with IL-6. 33 pathways contain TP53, such as MAPK signaling pathway and P53 pathways, etc. 100 target genes connect closely with TP53. 13 pathways contain VEGFA, such as the PI3K-Akt signal pathway. 100 target genes are most closely related to VEGFA. 41 pathways contain TNF, such as the hepatitis B signaling pathway and cancer proteoglycan pathway. And 99 target genes are most closely related to TNF. Molecular docking showed that the core targets IL-6, TP53, VEGFA, TNF, and MARK1 had a certain binding activity with the main active components such as quercetin, kaempferol, and isorhamnetin. AM-AS compound may improve oxidative stress, inhibiting inflammation, and reducing insulin resistance to treat diabetic glomerular disease and renal fibrosis by acting on IL-6, TP53, VEGFA, TNF, and other targets to regulating TNF signaling pathway, insulin resistance, tumor necrosis factor signaling pathway, etc. The possibility of treating DN with the AM-AS compound was preliminarily verified by molecular docking.

It was found that the AM-AS compound significantly reduces the level of insulin resistance and affects lipid metabolism in early atherosclerosis of diabetic rats^28^. AM and its extract can lower blood sugar levels, increasing glomerular filtration rate, reducing urinary protein, preventing glomerular mesangial thickening^29^, and delaying the progression of DN^30–32^. AS compound help reduces the expression of NF-κB, transform growth factor-β1(TGF-β1), glycosylation end products (AGEs), the blood glucose and urinary albumin excretion rate. AS has a certain renal protection effect, such as regulating creatinine clearance rate in diabetic rats^33^. Quercetin can improve renal function by reducing oxidative stress, the overexpression of TGF-β1, and connective tissue growth factor (CTGF) in renal tissue of diabetic rats^34^. Kaempferol decreases the expression of tumor necrosis factor-α (TNF-α), interleukin-1β(IL-1β), and TGF-β1 in NRK-52E cells induced by high glucose. Kaempferol restrains the processes of oxidative stress, inflammatory response, and fibrosis^35^, promotes insulin release^36^. By inhibiting the NF-κB signaling pathway, isorhamnetin interferes the expression of downstream TNF-α, IL-1β, IL-6, intercellular adhesion molecule-1 (ICAM-1), and TGF-β1, thereby alleviating the inflammatory response and oxidative stress^37^, improving fasting blood glucose and lipid metabolism, and protecting kidney^38^.

PI3K/Akt signaling pathway is closely related to glucose metabolism and is one of the downstream pathways of insulin. Overactivation of the PI3K/Akt signaling pathway can lead to structural damage and functional abnormalities of podocytes, which cause proteinuria^39,40^. VEGF interferes with angiogenesis and transformation of renal tubular epithelial cells to mesenchymal cells (EMT) by affecting PI3K/Akt signaling pathway^41^. TGF-β1 can stimulate cell proliferation and regulate ECM synthesis^42^, and the MAPK pathway can mediate the matrix deposition in the mesangial region. By blocking the TGF-β/MAPK/PPAR-γ pathway, the AM compound improves the blood glucose level, blood urea nitrogen (BUN), 24 h proteinuria, and other renal function indexes in diabetic rats^43,44^. HIF-1 plays a critical role in the prevention of early response to tissue damage caused by diabetes, and abnormal HIF-1 signaling pathways can accelerate kidney damage^45^. HIF can activate NF-κB^46^. The NF-κB signaling pathway is a transduction pathway of inflammatory signals, which is closely related to the development of DN^47^. Hypoxia can inhibit the activity of PHD1, leading to the activation of IKK, which in turn phosphorylates IκB, and leading to the transcriptional activation of downstream genes, such as inflammatory factors^48^. Our previous research found^6,7^ that the AM-AS compound can improve the related clinical symptoms of DN rats by regulating NF-κB and IL-6 related genes or pathways. Astragaloside IV combined with ferulic acid can intervene NF-κB, TNF-α pathway and improve the endothelial dysfunction of diabetic vascular disease. TNF signaling pathways are mainly related to immune function and inflammatory response. TNF-α can activate NF-κB, induce inflammatory cell infiltration, aggravate renal fibrosis, and eventually lead to renal injury^48^. It was found that the AM-AS compound could affect the concentration of TNF-α^28^, and their extracts inhibit the proliferation and fibrosis of glomerular mesangial cells (GMCs) in diabetic rats^49^. AM polysaccharides can reduce the levels of serum IL-6 and AngII in DM rats, and inhibit the expression of TGF-β1 and TNF-α^50^.

The results of in vivo study show that the AM-AS compound can inhibit collagen fiber proliferation and inflammation in diabetic rats. Combined with the results of molecular docking, we can speculate that AM-AS compound may act on key signal pathways such as TNF pathway, insulin resistance pathway, HIF-1 pathway, NF- kappa B pathway, and cAMP pathway through core targets such as TNF, IL-6, VEGFA, TP53, CASP3, JUN, AKT1 and so on. By this means, AM-AS compound can regulate the synthesis of NO, inhibit inflammatory reactions, oxidative stress, glycogen depositions, and collagen fiber formations, prevent abnormal morphologies of renal cells and dilatations of renal tubules, reduce urinary protein leakages, improve renal functions, and restraint renal functions damage caused by high glucose. The results of this study also suggest the accuracy of target prediction in network pharmacology.

## Conclusion

Through analysis of the main biologically active components of the AM-AS compound and their pharmacological mechanism, it is found that the AM-AS compound acts on multiple targets and proteins of multiple signal pathways, intervenes inflammation and oxidative stress delays the progression of DN. In the follow-up study, we plan to design animal pharmacological experiments in vivo and in vitro to conduct in-depth observations on the mechanism of the AM-AS compound in treating DN, to provide more references for its clinical application and development.
